# Genomic Characterization of *Bacillus safensis* Isolated from Mine Tailings in Peru and Evaluation of Its Cyanide-Degrading Enzyme CynD

**DOI:** 10.1128/aem.00916-22

**Published:** 2022-06-28

**Authors:** Santiago Justo Arevalo, Daniela Zapata Sifuentes, Andrea Cuba Portocarrero, Michella Brescia Reátegui, Claudia Monge Pimentel, Layla Farage Martins, Paulo Marques Pierry, Carlos Morais Piroupo, Alcides Guerra Santa Cruz, Mauro Quiñones Aguilar, Chuck Shaker Farah, João Carlos Setubal, Aline Maria da Silva

**Affiliations:** a Facultad de Ciencias Biológicas, Universidad Ricardo Palma, Lima, Peru; b Departamento de Bioquímica, Instituto de Química, Universidade de São Paulo, São Paulo, Brazil; Universidad de los Andes

**Keywords:** *Bacillus pumilus* group, bioremediation, core genome

## Abstract

Understanding the biochemistry and metabolic pathways of cyanide degradation is necessary to improve the efficacy of cyanide bioremediation processes and industrial requirements. We have isolated and sequenced the genome of a cyanide-degrading *Bacillus* strain from water in contact with mine tailings from Lima, Peru. This strain was classified as Bacillus safensis based on 16S rRNA gene sequencing and core genome analyses and named B. safensis PER-URP-08. We searched for possible cyanide-degradation enzymes in the genome of this strain and identified a putative cyanide dihydratase (CynD) gene similar to a previously characterized CynD from Bacillus pumilus C1. Sequence analysis of CynD from *B. safensis* and *B. pumilus* allow us to identify C-terminal residues that differentiate both CynDs. We then cloned, expressed in Escherichia coli, and purified recombinant CynD from *B. safensis* PER-URP-08 (CynD_PER-URP-08_) and showed that in contrast to CynD from *B. pumilus* C1, this recombinant CynD remains active at up to pH 9. We also showed that oligomerization of CynD_PER-URP-08_ decreases as a function of increased pH. Finally, we demonstrated that transcripts of CynD_PER-URP-08_ in *B. safensis* PER-URP-08 are strongly induced in the presence of cyanide. Our results suggest that the use of *B. safensis* PER-URP-08 and CynD_PER-URP-08_ as potential tool for cyanide bioremediation warrants further investigation.

**IMPORTANCE** Despite being of environmental concern around the world due to its toxicity, cyanide continues to be used in many important industrial processes. Thus, searching for cyanide bioremediation methods is a matter of societal concern and must be present on the political agenda of all governments. Here, we report the isolation, genome sequencing and characterization of cyanide degradation capacity of a bacterial strain isolated from an industrial mining site in Peru. We characterize a cyanide dehydratase (CynD) homolog from one of these bacteria, Bacillus safensis PER-URP-08.

## INTRODUCTION

Cyanide is a highly toxic compound used in several industrial processes (1) given its capacity to form tight complexes with different metals (2–4). Sublethal doses of cyanide in the environment have negative effects on physiological processes such as osmoregulation, early development, growth, fat gain, and spermatogenesis of vertebrates and invertebrates (5). Industries that generate cyanide-containing wastes must reduce its concentration before discarding them to the environment and, as such, proper strategies have to be implemented for cyanide remediation (6). These strategies include photodecomposition, chemical oxidation, volatilization, microbial oxidation, hydrolysis, and precipitation of metalocomplexes (2).

Cyanide bioremediation by bacteria that express nitrilases with the capacity to degrade cyanide is one possible low-cost and environmentally friendly approach (2). Nitrilases are a superfamily of proteins characterized by a tertiary structure consisting of an alpha-beta-beta-alpha fold and a dimer as a basic unit. This superfamily has been divided into 13 branches, with branch one corresponding to enzymes that cleave the nitrile group into ammonia and its respective carboxylic acid. The other 12 branches are structurally similar, though their catalytic activities do not involve cleavage of nitriles but instead correspond to amidases, carbamylases, and N-acyl transferases (7).

Two types of cyanide-degrading enzymes perform their activities through a hydrolytic pathway: cyanide hydratases (CHTs) and cyanide dihydratases (CynDs). CHTs convert cyanide into formamide, consuming one water molecule in the reaction, whereas the reaction catalyzed by CynDs consumes two water molecules and generates formic acid and ammonia. Both CHTs and CynDs typically form large multisubunit helical aggregates (8).

Only the CynDs from *B. pumilus* C1, Pseudomonas stutzeri AK61 and Alcaligenes xylosoxidans DF3 have been experimentally tested (9–11). CynD from Bacillus pumilus C1 (CynD_C1_) and from P. stutzeri AK61 (CynD_AK61_) form oligomers of 18 and 14 subunits, respectively (12, 13). Furthermore, wild-type CynD_C1_ and CynD_AK61_ are active at up to pH 8 (14, 15).

Several *Bacillus* species have been shown to degrade cyanide using different enzymes. These include, for example, the enzyme B-cyanoalanine synthase in Bacillus megaterium (16), gamma-cyano-alpha-aminobutyric acid synthase in *B. stearothermophilus* (17), rhodanase in Bacillus cereus (18), and CynD in Bacillus pumilus (9). On the other hand, some other cyanide-degrading *Bacillus* species degrade cyanide through uncharacterized enzymes (19–21).

The Bacillus pumilus group consists mainly of three species: Bacillus altitudinis, Bacillus safensis, and Bacillus pumilus. These three species share more than 99% sequence identity in their 16S rRNA gene (22), hampering taxonomic classification based solely on this locus. Studies using multiple phylogenetic markers have demonstrated that ~50% of the Bacillus pumilus group genomes deposited in the NCBI database could be misclassified (23).

It is plausible to speculate that CynDs isolated from *Bacillus* strains from diverse environments could present different properties, some of which could be better suited for certain industrial applications. Therefore, the characterization of CynD homologs from other species can expand our understanding on the functioning and plasticity of this enzyme. Furthermore, some aspects of the biology of this enzyme have not been thoroughly studied. For instance, the oligomeric state of CynD is strongly pH dependent (12); however, the effect on oligomerization at pHs greater than 9 has not been reported. Also, it is unknown whether CynD is constitutively expressed in basal metabolism or is part of a specific physiological response, for instance, induced by the presence of cyanide.

Here, we describe the isolation of an indigenous *Bacillus* strain from a Peruvian mine tailing site and the sequencing of its genome. We investigated its phylogenetic relationship with other species of the Bacillus pumilus group. We identified a gene coding for a cyanide dihydratase (CynD) that is most likely the enzyme responsible for cyanide degradation in this selected strain. A recombinant CynD was expressed and purified, its catalytic parameters were determined, and the quaternary structure was studied at different pHs. We also demonstrated that CynD transcripts are strongly induced in the presence of cyanide.

## RESULTS AND DISCUSSION

### A *Bacillus* isolate with cyanide degradation capacity.

**FIG 1:**
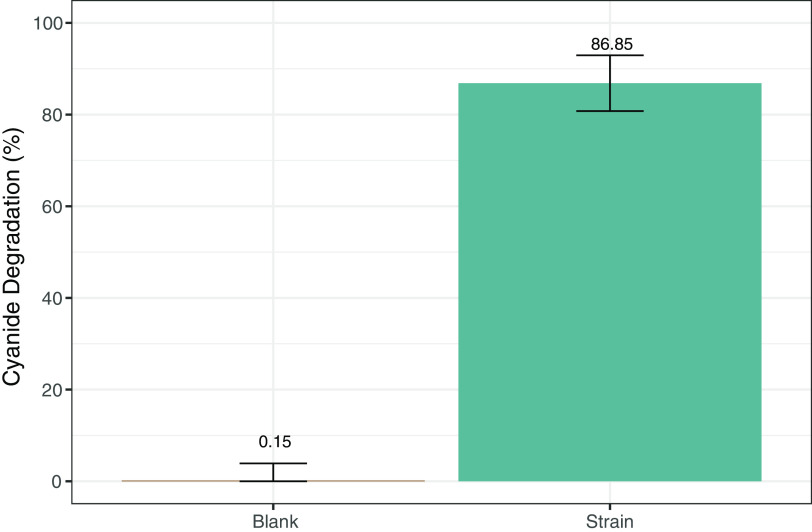
Cyanide degradation by the strain 8 (then named *B. safensis* PER-URP-08). The strain 8 was incubated with 3.84 mM NaCN for 4 h. After this time, remaining cyanide was quantified using the picric acid method. As a control, cyanide degradation in the same condition but in the absence of bacterial cells was also measured (blank). The value and the error bars represent the means and the standard deviations, respectively, of three replicates.

Several colonies were obtained from a sample of water in contact with mine tailing from a river near Casapalca and La Oroya mines located in San Mateo de Huanchor, Lima, Peru (see Fig. S1 in the supplemental material). After selection in cyanide containing medium, 20 individual isolates were further screened for the ability to degrade cyanide. One of the isolates (strain 8) exhibited the greatest efficiency to degrade cyanide (see Table S2), degrading ~86% of the cyanide in 4 h (initial concentration = 3.84 mM) (Fig. 1). This strain was selected for further studies.

Sequencing of the V6, V7, and V8 variable regions of 16S rRNA gene of the selected isolate (isolate number 8), and BLAST analysis showed that it belongs to the genus *Bacillus* (see Table S3). Isolate 8 was classified as a member of the Bacillus pumilus group based on the 16S rRNA gene sequence (see Table S3) and was provisionally named *Bacillus* sp. strain PER-URP-08.

The genome of *Bacillus* sp. PER-URP-08 was then sequenced in order to obtain a more accurate taxonomic classification, as well as to gain insights about possible routes of cyanide degradation. Table S4 provides a summary of assembly and annotation metrics of the *Bacillus* sp. PER-URP-08 genome.

### *Bacillus* sp. PER-URP-08 is classified as *Bacillus safensis* based on core-genome comparisons.

We performed a genome-wide comparative analysis of *Bacillus* sp. PER-URP-08 with 132 genomes of species from the *B. pumilus* group retrieved from the GenBank/NCBI database (24) and identified 1,766 coding sequences present in all the genomes (core genes). An identity matrix based on an alignment of these core genes showed three well-defined branches and two genomes that do not belong to any of these three branches (see Fig. S2).

**FIG 2:**
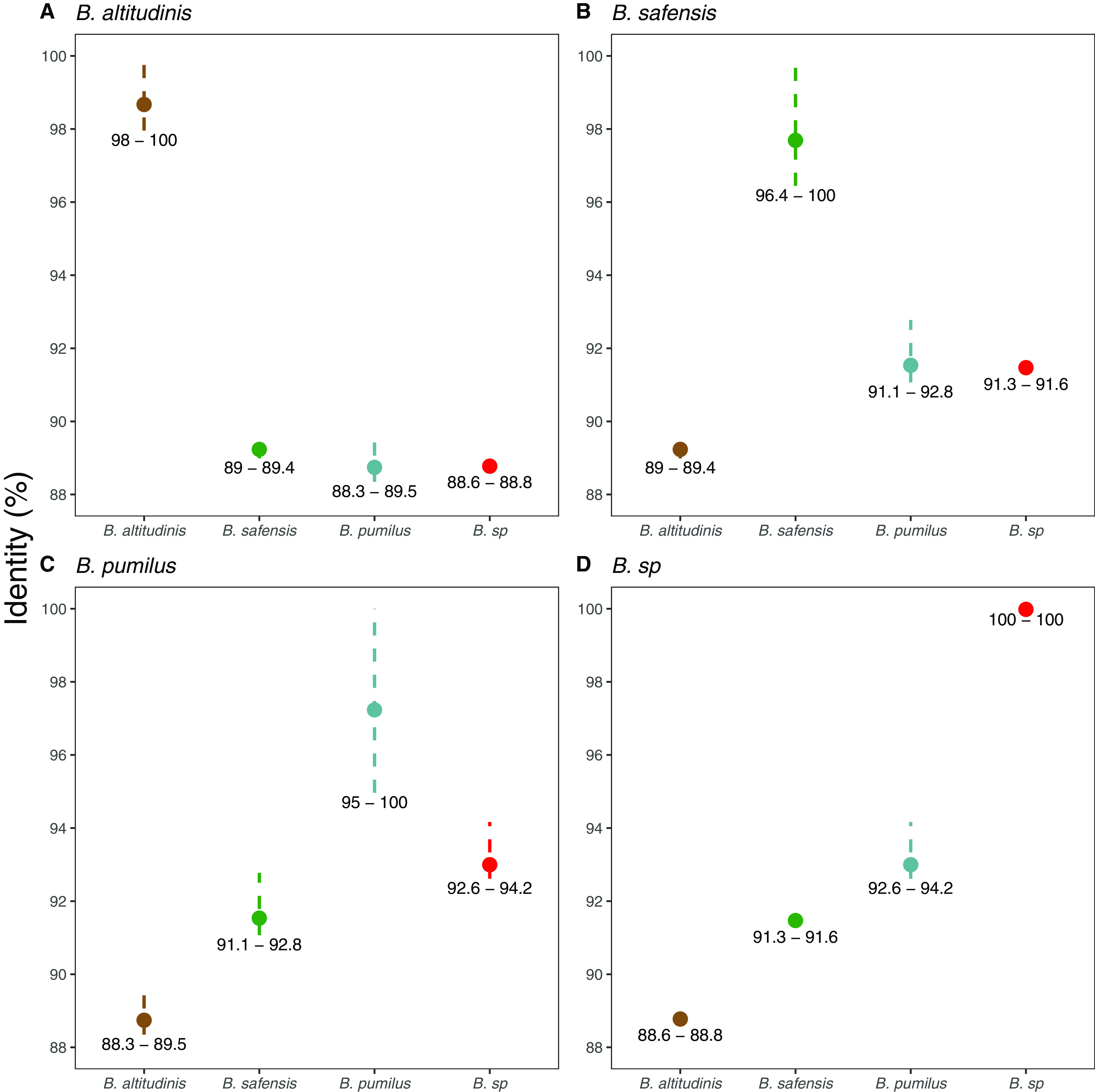
Range of identities between *Bacillus* species inside the Bacillus pumilus group. (A to D) Plots showing the range of identity when comparing *B. altitudinis* (A), *B. safensis* (B), *B. pumilus* (C), or *Bacillus* sp. (D) core genomes with itself or with other groups.

Branch 1 (see Fig. S2, names in brown) contains several strains already characterized as Bacillus altitudinis by different methods (for instance, BA06, ku-bf1, and B-388 [25]). The core genes within the *B. altitudinis* branch share more than 98% identity whereas they share less than 89.5% identity with core genomes of the other two branches (Fig. 2A).

Identity of core genes in branch 2 is greater than 96%, and this branch is more related to branch 3 (*B. pumilus* [see below]) than to branch 1 (*B. altitudinis*) (Fig. 2B). Branch 2 (see Fig. S2, names in green) contains the Bacillus safensis type strain FO-36b (26), as well as other strains already classified as *B. safensis* such as B4107, B4134, and B4129 (23). *Bacillus* sp. PER-URP-08 clustered with this branch, showing greatest similarity to the type strain FO-36b (99.2% identity) (see Fig. S2), and was therefore named Bacillus safensis PER-URP-08.

Branch 3 (see Fig. S2, names in blue) contains the SAFR-032 strain that was the first completely sequenced genome of *B. pumilus* (27, 28). This branch appears to be more heterogeneous than the other two branches (*B. altitudinis* and *B. safensis*) with a little more than 95% identity of the core genes of this branch (Fig. 2C). In addition, two genomes isolated from Mexico (CH144a_4T and 145) share less than 95% identity with branch 3 and even less with branches 1 and 2 (Fig. 2D). The fact that these two genomes share less than 95% identity with all the other genomes in the analysis (Fig. 2D) indicates that CH144a_4T and 145 strains should be classified as different species outside the *B. pumilus* group. Table S5 summarizes the proposed classification of the strains of the *B. pumilus* group analyzed here.

### A cyanide dihydratase homolog is codified by *B. safensis* PER-URP-08.

To gain insight regarding the enzymes likely responsible for cyanide degradation in *B. safensis* PER-URP-08, we first searched for genes coding for proteins related to nitrilases. The PFAM database annotates homologs of nitrilases as CN_hydrolases under the PFAM code PF00795. Using IMG/M system tools (29), we determined the presence of three proteins containing CN_hydrolase domains in *B. safensis* PER-URP-08: EGI07_01665 (*yhcX*), EGI07_17510, and EGI07_08135 (CynD).

YhcX is probably involved in the degradation of indole 3-acetonitrile, a subproduct of tryptophan metabolism (30), while EGI07_17510 is a protein of unknown function. CynD homologs (see Fig. S3) are predicted to hydrolyze cyanide to produce ammonia and formic acid (2, 31). We therefore carried out experiments to test the hypothesis that *B. safensis* PER-URP-08 CynD is capable of cyanide degradation.

### C-terminal residues differentiate CynDs from *B. pumilus* and *B. safensis*.

**FIG 3:**
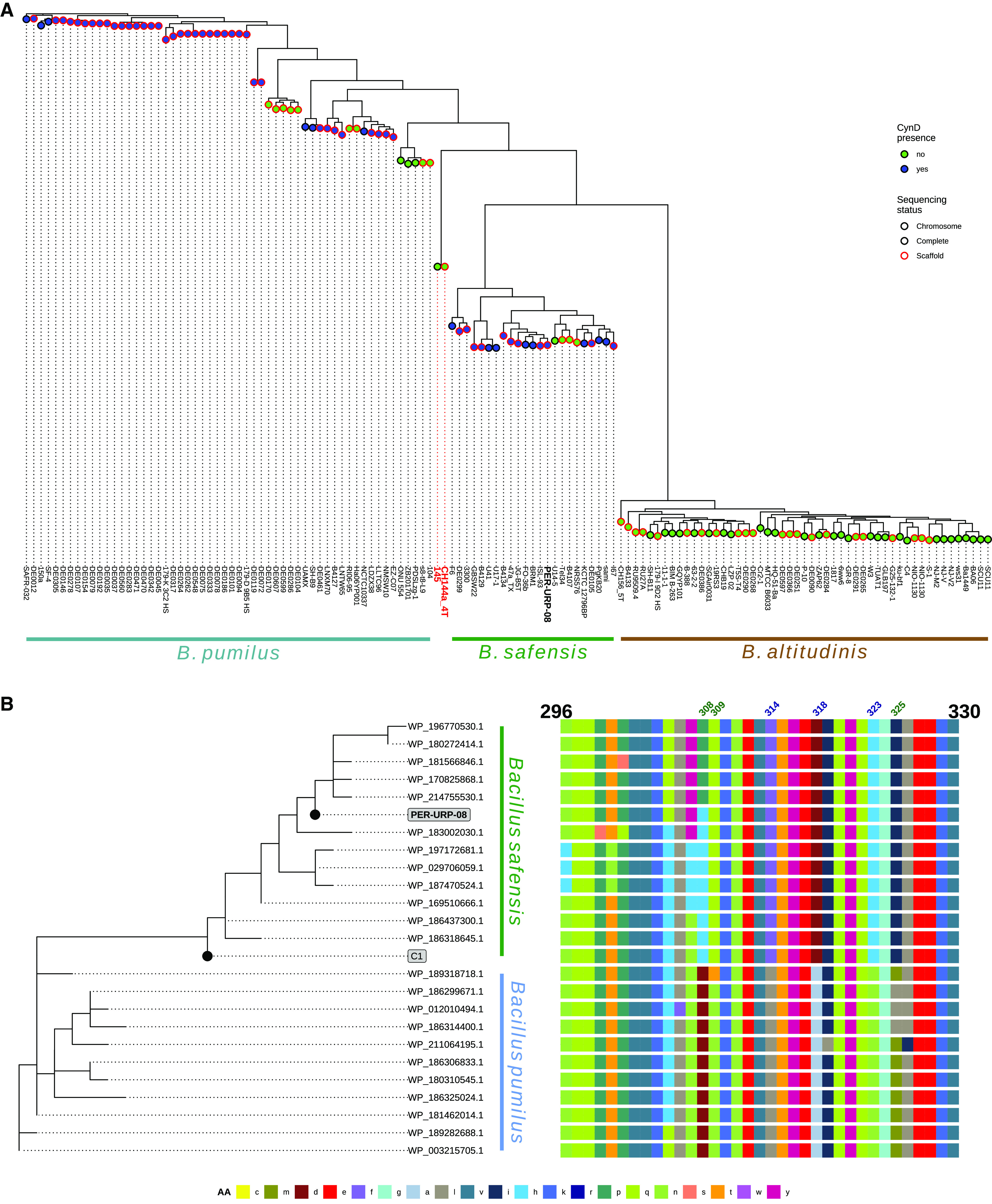
CynD is present in some genomes of *B. pumilus* and *B. safensis.* (A) Maximum-likelihood tree of core genomes of 132 Bacillus pumilus group strains showing separation between three species. The colors of the circles represent the absence (green) or presence (blue) of CynD homologue in the genome. Circles with black and red borders represent complete genomes (“chromosome” or “complete” sequencing status in NCBI) and possibly not complete genomes (“scaffold” sequencing status in NCBI). (B, left) Maximum-likelihood tree of full-length CynD sequences. (Right) Alignment of the respective C-terminal regions (residues 296 to 330) of the CynD proteins. Numbers indicate the positions that are completely conserved in Bacillus safensis (blue) or *B. pumilus* (green).

We first constructed a maximum-likelihood (ML) phylogenetic tree based on the 132 core genomes of strains from the *B. pumilus* group (Fig. 3A; see also Table S5) and searched for orthologs of CynD in the strains present in the ML tree (see Materials and Methods for details of the search). The ML tree confirmed the three branches identified above (Fig. 2; see also Fig. S2) and that same two genomes (CH144a_4T and 145) do not belong to any of these branches (Fig. 3A). Intriguingly, CynD-encoding sequences were found in some representatives of *B. pumilus* (44 of 56) and *B. safensis* (19 of 23) but not in *B. altitudinis*. Three monophyletic *B. pumilus* and one monophyletic *B. safensis* clades lack CynD (Fig. 3A). This could be due to processes of gene gain and/or loss in the strains, and further studies are necessary to distinguish between these or other possibilities. It is also possible that some *cynD* genes were not sequenced in some incomplete genomes.

Next, we identified 23 different sequences of CynD in the 132 genomes (see Table S6) and a ML phylogenetic tree based on these amino acid sequences was constructed, including the sequences of the CynD from strain C1 (CynD_C1_; accession number AAN77004.1) and of the CynD from *B. safensis* PER-URP-08 (CynD_PER-URP-08_). A clear separation between CynD from *B. safensis* and from *B. pumilus* could be observed in the ML tree (Fig. 3B). Interestingly, CynD_C1_ appears more closely related to the *B. safensis* group (Fig. 3B). Due to the several taxonomic misclassifications of strains belonging to the *B. pumilus* group (as reported here and by others [22, 23, 25]), it is likely that strain C1 truly belongs to a *B. safensis* species; however, the complete genome of C1 is not available to confirm this hypothesis.

The most variable region in the nitrilase protein family is the C-terminal region (8, 32). Thus, we mapped a phylogenetic tree obtained from the full-length sequences of identified CynDs homologs to an alignment of the C-terminal region (residues 296 to 330) (Fig. 3B). Residues F314, D318, and H323 in CynD proteins from the *B. safensis* group are L314, A318, and N323 in CynD proteins from the *B. pumilus* group. Other residues can vary in one of the species but are strictly conserved in the other; for instance, residues Q309 and I325 in *B. safensis* are T309 or N309 and M325 or L325 in *B. pumilus*. Residue 308 can be P or M in *B. safensis* but is strictly D in *B. pumilus* (Fig. 3B). The CynD_C1_ had the amino acids strictly conserved in *B. safensis*, supporting the conclusion that the C1 strain belongs to the *B. safensis* species. Furthermore, residue 27, outside the C-terminal region, is E in *B. safensis* and strain C1 but Q in *B. pumilus*.

### CynD from *B. safensis* PER-URP-08 is still active at pH 9.

**FIG 4:**
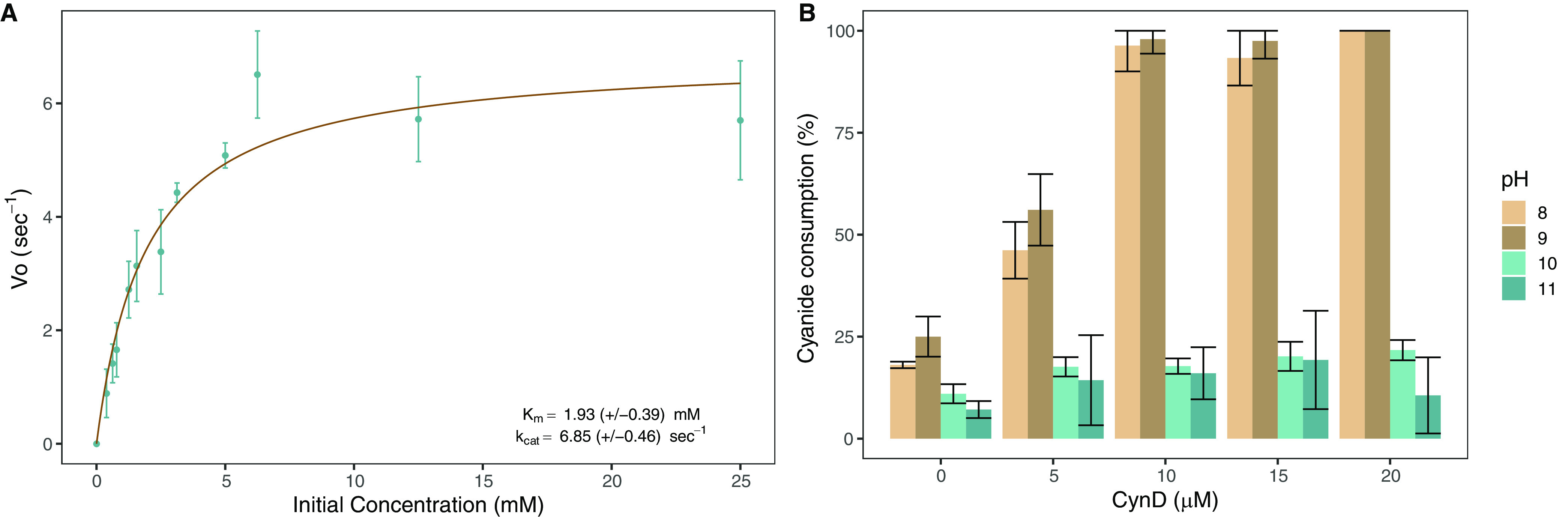
Enzyme kinetics and pH dependence of CynD_PER-URP-08_. (A) Plot of CynD_PER-URP-08_ initial velocity (*V*_0_) versus initial concentration of cyanide adjusted to the Michaelis-Menten equation. *K_m_* and *k*_cat_ constants were calculated assuming no cooperativity. Reactions were carried out with 500 nM CynD_PER-URP-08_ in 100 mM NaCl–20 mM Tris-HCl (pH 8.0) at 30°C. (B) Percentage of cyanide consumption at different pHs using different CynD_PER-URP-08_ concentrations (5 to 20 μM) and an initial cyanide concentration of 36 mM. Reaction time, 10 min.

We then went on to characterize some biochemical properties of CynD_PER-URP-08_. First, we cloned, expressed, and purified recombinant CynD_PER-URP-08_ in E. coli (see Fig. S4) and determined its basic kinetic constants. Although CynDs are known to be able to adopt different oligomeric states, no evidence of cooperativity was observed in our enzymatic assays (Fig. 4A). Instead, a simple Michaelis-Menten model fits the experimental data adequately. At 30 °C and pH 8, the *K_m_* and *k*_cat_ values estimated using this model were 1.93 ± 0.39 mM and 6.85 ± 0.46 s^−1^, respectively (Fig. 4A; see also Fig. S5).

Due to the volatility of hydrogen cyanide in its protonated (HCN) state and its pKa of 9.2 (33), the bioremediation processes should be carried out at or above pH 9. To test whether CynD_PER-URP-08_ is active at pHs greater than 8, we tested its activity at pH 9, 10, and 11. Figure 4B shows that different concentrations of recombinant CynD_PER-URP-08_ carrying a C-terminal 6×His tag showed considerable activity at pH 8 and 9 but not at pH 10 and 11.

Other wild-type CynDs have been shown to be active only up to pH 8 (14, 15) and CynD_C1_ with C-terminal 6×His tag had its activity compromised at pH 9 (34). The CynD_C1_ and CynD_PER-URP-08_ sequences only differ at five positions: I18V, S25T, E155D, H305Q, and N307Y, respectively, with the last two substitutions H305Q and N307Y near the C terminus.

Other studies were able to generate active versions of CynD active at pH 9 by introducing mutations in some conserved positions (K93R, Q86R, E96G, D254G) or by replacing the C-terminal region from CynD_C1_ with the C-terminal region from CynD from Pseudomonas stutzeri (35, 36). It is worth noting that wild-type CynD from P. stutzeri has not been tested at pH 9.

### Alkaline pH reduces the degree of oligomerization of CynD_PER-URP-08_.

The oligomerization state of nitrilases have been associated with enzyme activity and stability (14, 35–39). In CynD_C1_ and CynD from P. stutzeri, mutations in the C-terminal region decrease oligomerization (14, 36, 37). The C-terminal region of nitrilases stabilizes the spiral structure through criss-crossed beta sheets in the center of the oligomer (8, 40). Also, Acidic pH has been shown to promote higher-order oligomerization states of CynDs (12, 36); however, the effects of pHs greater than 9 have not been reported.

**FIG 5:**
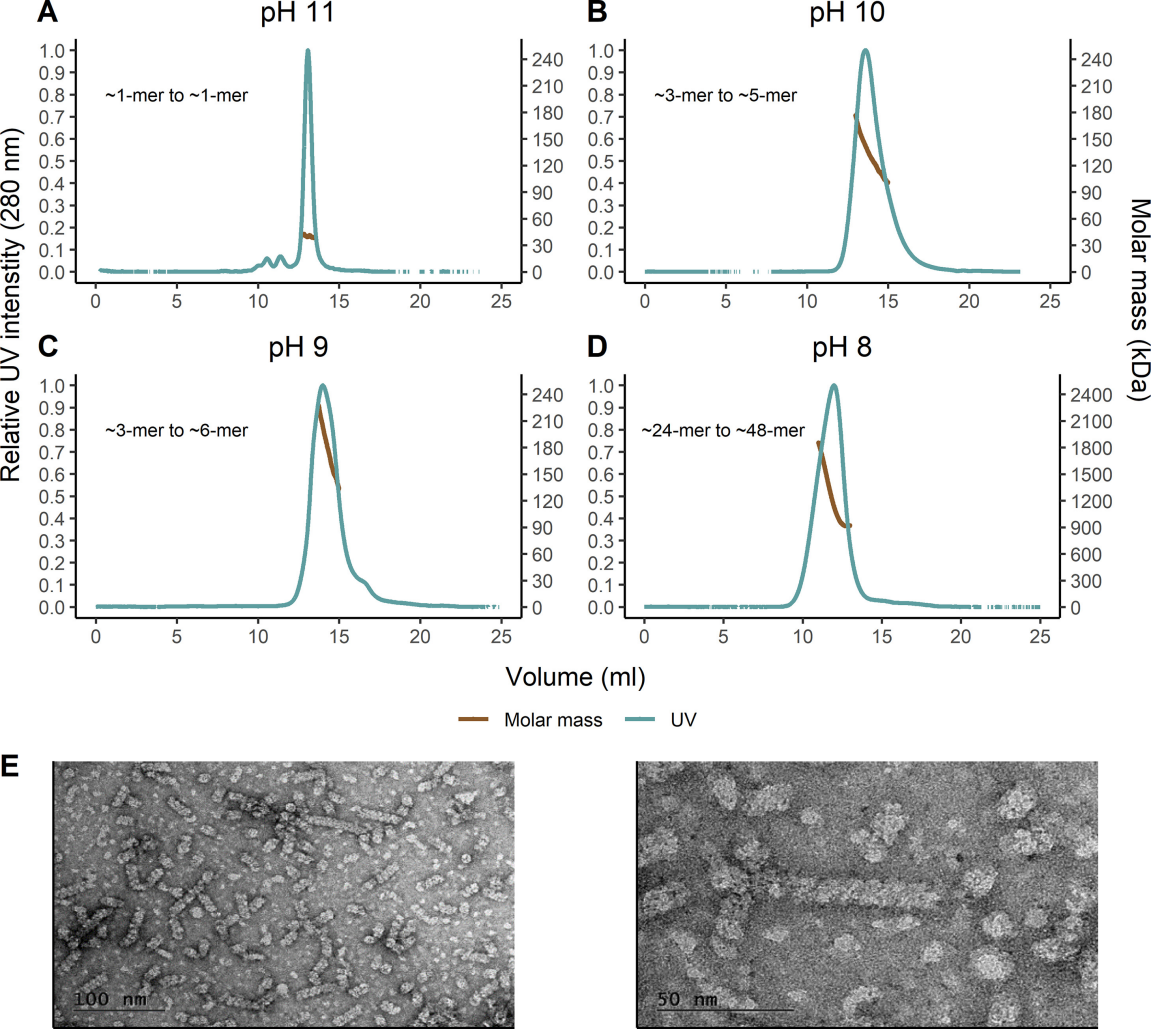
pH-dependent oligomerization of CynD_PER-URP-08_. (A to D) SEC-MALS analysis of CynD as a function of pH. Plots of the UV intensity/molar mass for CynD_PER-URP-08_ at different pHs are shown. (E) Transmission electron micrographs of CynD_PER-URP-08_ at pH 8 at two different magnifications (50,000×, right; 100,000×, left) reveal helical structures of variable lengths.

Since CynD_PER-URP-08_ has differences in the C-terminal region with respect to other CynDs, we used SEC-MALS (size exclusion chromatography coupled to multiangle light scattering) to compare the oligomerization states of CynD_PER-URP-08_ at different pHs. As expected, pHs higher than 8 result in lower-molecular-weight oligomers. At pH 11, the monomer (38.5 kDa) is the predominant species (Fig. 5A), whereas pH 10 and 9 presented oligomeric states ranging from ~3-mer to ~5-mer (pH 10, 100.85 to 176.34 kDa) and ~4-mer to ~6-mer (pH 9, 133.19 to 226.99 kDa) (Fig. 5B and C). At pH 8, CynD_PER-URP-08_ presented oligomers ranging from ~24-mer to ~48-mer (918.31 to 1,851.39 kDa; Fig. 5D) in contrast to what was reported for CynD_C1_ at pH 8, which forms an 18-mer spiral (12). These differences could be due to differences in amino acid sequence between CynD_PER-URP-08_ and CynD_C1_ or due to the presence of the C-terminal 6×His tag in CynD _PER-URP-08_. Experiments with CynD_C1_ were carried out with untagged protein or with protein carrying an N-terminal 6×His tag (12, 35, 36, 39). Electron micrographs of negatively stained CynD_PER-URP-08_ at pH 8 showed spirals of different sizes, supporting the conclusion that CynD_PER-URP-08_ at this pH adopts a range of different oligomerization states (Fig. 5E).

### Expression of CynD_PER-URP-08_ from *B. safensis* PER-URP-08 is induced in the presence of cyanide.

**FIG 6:**
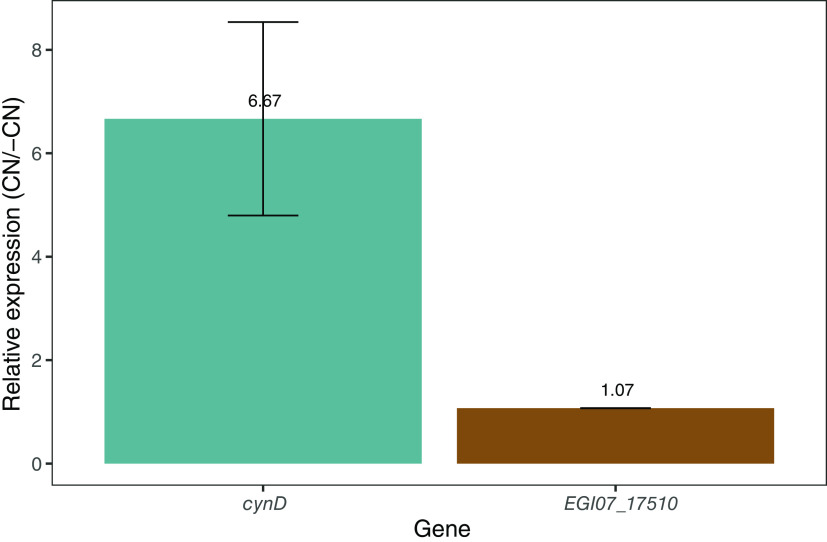
*cynD*_PER-URP-08_ but not EGI07_17510 is induced in the presence of cyanide. The relative expression measured by RT-qPCR showed that when Bacillus safensis is exposed to cyanide, the RNA levels of *cynD* are 6.67-fold greater than in the absence of cyanide. In contrast, the expression of another nitrilase gene (*EGI07_17510*) is the same in presence or absence of cyanide.

Some previous studies have considered the possibility that CynD gene expression is regulated by cyanide, but this point remains unclear (12). To address this question, we exposed *B. safensis* PER-URP-08 to 3.85 mM NaCN (equivalent to 100 ppm CN^–^ or 188 ppm NaCN) at 30°C for 4 h without agitation. The mRNA levels of *cynD* were subsequently measured and compared to the levels observed in cells grown in the absence of CN^–^. We observed a 6.7-fold increase in the expression of *cynD* in the presence of cyanide (Fig. 6). To evaluate whether this overexpression is specific for *cynD* nitrilase and not to other nitrilases of *B. safensis* PER-URP-08, we also measured the mRNA levels of EGI07_17510 that also possesses a CN_hydrolase domain. We did not observe differences in EGI07_17510 expression in the presence or absence of cyanide. To our knowledge, this is the first report showing induction of the expression of *cynD* in the presence of cyanide. This could possibly be a physiological response of the bacteria in order to protect itself from the toxic effects of the compound, but further studies are necessary to more fully understand the molecular mechanisms behind this response.

### Conclusions.

Here, we report the isolation and the genome sequence of a cyanide-degrading *Bacillus* strain obtained from water in contact with mine tailings in Lima, Peru. It was phylogenetically classified and named Bacillus safensis PER-URP-08. Comparative genomic analyses indicate that some strains currently classified as *B. pumilus* with publicly available genomes should be reclassified as Bacillus altitudinis (strains TUAT1, MTCB 6033, SH-B11, and C4). Furthermore, we propose that strains CH144a_4T and 145 should be classified as belonging to a new species distinct from *B. pumilus*, *B.safensis*, or *B. altitudinis*. We propose that, in *B. safensis* PER-URP-08, EGI07_08135 codes for an ortholog of cyanide dihydratase CynD that imparts the cyanide degradation ability to this strain.

CynD orthologs were found in *B. pumilus* and *B. safensis*, but not in *B. altitudinis*. Despite the high identity (>97%) of CynDs, conserved differences in the C terminus allow us to differentiate between CynD from *B. safensis* or *B. pumilus* (at least in the analyzed genomes). In addition, sequence analysis of the previously described CynD from strain C1 (CynD_C1_), referred to as *B. pumilus* CynD in the literature, showed that it is more closely related to CynDs from *B. safensis* than from *B. pumilus*.

We characterized some aspects of CynD from *B. safensis* PER-URP-08 (CynD_PER-URP-08_). Our results corroborate results previously described for CynDs from other species and add new knowledge about these enzymes. First, enzymatic assays with CynD_PER-URP-08_ detected no evidence of cooperativity despite the known oligomerization patterns of these enzymes. Second, the *K_m_* and *k*_cat_ values of CynD_PER-URP-08_ were 1.93 mM and 6.65 s^−1^, respectively. Third, despite the fact that CynD_PER-URP-08_ and CynD_C1_ only differ in five positions, CynD_PER-URP-08_ retains almost the same activity in pH 9 as that observed at pH 8, whereas CynD_C1_ has been reported to be almost inactive at pH 9. Fourth, as pH is known to influence the oligomerization of CynDs, we show that at pH 8, CynD_PER-URP-08_ forms spirals made up of an estimated ~24 to ~48 subunits, indicating that several oligomeric states are present in this pH and slightly larger than the 18-mer oligomers previously reported for CynD_C1_ at this pH. Moreover, at pH 11, the CynD_PER-URP-08_ monomer was observed. Finally, we showed for the first time that the abundance of CynD_PER-URP-08_ transcripts increased 6-fold when bacterial cells were exposed to CN^–^.

Altogether, the results we report here suggest that further investigations to explore the potential of *B. safensis* PER-URP-08 and CynD_PER-URP-08_ for cyanide bioremediation are warranted.

## MATERIALS AND METHODS

### Isolation of cyanide-resistant strains.

Water in contact with mine tailings was collected from a river near Casapalca and La Oroya mines located in San Mateo de Huanchor, Peru (latitude, −11.4067; longitude, −76.3361 at 4221 MASL) (see Fig. S1). The samples were collected in 2-L sterile bottles and transported at 4°C. Portions (100 mL) of the sample were added to Erlenmeyer flasks containing 20 mL of sodium carbonate (21 g/L), sodium bicarbonate (9 g/L), sodium chloride (5 g/L), and 0.5 potassium nitrate (g/L). The cell suspensions were maintained for 12 h at 37°C; then, a 1-mg/L final concentration of cyanide in the form of sodium cyanide was added, and each Erlenmeyer flask was sealed with a cap. The cell suspensions were maintained for another 24 h at 37°C. Samples of the medium were streaked in petri dishes with nutrient agar (5 g/L peptone, 5 g/L yeast extract, 5 g/L sodium chloride, and 1% agar), followed by incubation at 37°C for 24 h. Single colonies were isolated, grown in nutrient broth (5 g/L peptone, 5 g/L yeast extract, and 5 g/L sodium chloride), supplemented with 20% glycerol, and stored at −80°C.

### Cyanide degradation assays of isolated strains.

The strains stored at −80°C were reactivated by streaking a sample on 2×TY (16 g/L tryptone, 10 g/L yeast extract, and 5 g/L NaCl) agar plates and growth at 37°C. Single colonies were then used to inoculate 5 mL of fresh 2×TY broth, followed by incubation at 37°C overnight at 100 rpm. Next, a 1:200 dilution of the culture was prepared in 5 mL of meat broth (1 g/L meat extract, 2 g/L yeast extract, 5 g/L peptone, and 5 g/L NaCl) and supplemented with 10 mg/L MnCl_2_. Meat broth and MnCl_2_ have been reported to stimulate the cyanide-degrading capacity of Bacillus pumilus (41). After 12 h of growth at 37°C (optical density at 600 nm = 1.5), 1 mL of the culture was transferred to a 1.5-mL capped tube, followed by centrifugation at 6,000 × *g* for 2 min. The pellet was resuspended in 1 mL of 3.84 mM NaCN (equivalent to 100 ppm [CN^–^] or 188 ppm NaCN) and incubated at 30°C for 4 h. The cell suspension was centrifuged at 6,000 × *g* for 2 min, and 25 μL of the supernatant was transferred to a 96-well microplate. Immediately after, 50 μL of 0.5% picric acid in 0.25 M sodium carbonate was added, and the mixture was heated for 6 min at 99°C (42). Finally, the absorbance at 520 nm was measured and compared to a standard curve of NaCN. A 1.5-mL capped tube with 1 mL of 3.84 mM NaCN without bacterial cells was used as a negative control. Degradation assays were performed in triplicate.

### Strain identification by 16S rRNA gene sequencing.

To determine the bacterial genera and/or species of the selected isolated strain, we used a fresh culture in nutrient agar. Five colonies were transferred to 50 μL of Milli-Q water and then heated to 100°C for 3 min in a dry bath. The samples were centrifuged at 10,000 × *g* for 5 min and the supernatant was used as a template to amplify a fragment that includes the V6, V7, and V8 variable regions of 16S rRNA gene (43). Next, 1 μL of the template plus 25 pmol of F_primer (5′-GCACAAGCGGTGGAGCATGTGG-3′) and R_primer (5′-GCCCGGGAACGTATTCACCG-3′) were mixed with 1× *Taq* buffer, 1.5 mmol of MgCl_2_, 0.2 mmol of each deoxynucleoside triphosphate, and 1 U of *Taq* DNA polymerase (Thermo Fisher Scientific) in a final reaction of 25 μL. The amplification program was an initial denaturation at 94°C for 5 min, followed by 30 cycles at 94°C for 45 s, 50°C for 45 s, and 72°C for 1 min, with a final extension of 10 min at 72°C. Next, 5 μL of the final reaction was used as a template for the sequencing reaction. The sequencing reaction was carried out using a BigDye terminator v3.1 cycle sequencing kit (Thermo Fisher Scientific) consisting of a 1× sequencing buffer, 25 pmol of F_primer or R_primer, and 2 μL of BigDye in a final volume of 20 μL. The program used was an initial denaturation at 94°C for 5 min, followed by 40 cycles at 94°C for 30 s, 50°C for 30 s, and 60°C for 4 min. After the sequencing reaction, 80 μL of 70% isopropanol was added, and the reaction tube was centrifuged at 4,000 × *g* at 4°C for 40 min. The supernatant was then discarded, and the sample was resuspended in 20 μL of Milli-Q water and injected into an ABI Prism 3130XL genetic analyzer (Thermo Fisher Scientific). The sequence obtained was used to perform BLASTn (44) searches against the GenBank/NCBI database (24) to identify the most similar sequences.

### Genome sequencing, assembly, and annotation.

The bacterial culture was grown in 2×TY broth (tryptone, 16 g/L; yeast extract, 10 g/L; and NaCl, 5 g/L) at 37°C for 18 h at 200 rpm. Genomic DNA purification was performed using a Wizard Genomic DNA purification kit (Promega). The DNA integrity was evaluated using 1% agarose gel electrophoresis gels stained with SYBRSafe (Invitrogen) and in a 2100 Bioanalyzer using an Agilent DNA 12000 chip. The DNA concentration and purity were estimated by using a NanoDrop One/OneC Microvolume UV-Vis spectrophotometer (Thermo Fisher Scientific). A shotgun genomic library was prepared using Nextera DNA Library Prep (Illumina) with a total DNA input of 20 to 35 ng. The resulting indexed DNA library was cleaned up with Agencourt AMPure XP beads (Beckman Coulter), and fragment sizes within the range of 200 to 700 bp were verified in a 2100 Bioanalyzer using an Agilent high-sensitivity DNA chip. Fragment library quantification was performed using a KAPA library quantification kit. The genomic library was subjected to a run using an Illumina MiSeq reagent kit v2 (2 × 250 cycles). Raw paired-end reads were assembled with Discovar (v52488) (45). This software has adapter trimming and read quality checking as part of its respective assembly processes. The tool Medusa (46) was used to generate the final genome scaffold using a set of five reference genomes (see Table S1). The final genome assembly was submitted to the IMG/M (29) and to the NCBI (24, 47) for automatic annotation.

### Phylogenetic analyses and identification of nitrilases.

Annotated genomes belonging to Bacillus pumilus, Bacillus safensis, or Bacillus altitudinis species in the category of “Chromosome,” “Scaffold,” or “Complete” were downloaded from the GenBank/NCBI (24). Using the software cd-hit (48, 49), we identified coding sequences that are not duplicated and present in all the genomes (core genes). A total of 1,766 core genes with more than 80% identity and at least 90% coverage were used in the analysis. Core genes were aligned using MAFFT with the FFT-NS-2 algorithm (50). The resulting alignments were concatenated and used to calculate a distance matrix based on identity using Biopython (51). Phylogenetic inference by maximum likelihood was done using the concatenated alignments as the input and IQ-TREE2 (52) with the evolution model GTR+F+R3, ultrafast bootstrap 1000 (53), and 1000 initial trees.

IMG/M tools (29) were used to identify nitrilases genes in the annotated genomes. Genes encoding the CN_hydrolase domain (PFAM code PF00795) were selected and checked regarding the genomic context and the related literature.

### Analysis of CynD sequences from *Bacillus pumilus* group genomes.

Protein sequence annotations from genomes belonging to Bacillus pumilus, Bacillus safensis, or Bacillus altitudinis in the category of “Chromosome,” “Scaffold,” or “Complete” were downloaded from GenBank/NCBI (24) and used to construct a local database. We ran a BLASTp search (44) using the query sequence AAN77004.1 against the constructed local database, and sequences with more than 90% identity and 100% coverage were identified as CynD orthologs. These sequences were aligned using MAFFT with the L-INS-I algorithm (50). The resulting alignment was used as an input for the phylogenetic inference by maximum likelihood using IQ-TREE2 (52) with the evolution model JTTDCMut+I (54), ultrafast bootstrap 1000 (53), 1000 initial trees, and the -allnni option.

### Cloning, expression, and purification of CynD.

The coding sequence for CynD was amplified from genomic DNA of strain PER-URP-08 using the primers (restriction sites are indicated in uppercase) F_CynD (5′-tttCATATGatgacaagtatttacccgaagtttc-3′) and R_CynD (5′-tttCTCGAGcactttttcttcaagcaaccc-3′) and cloned in the NdeI and XhoI sites of the pET-28 expression plasmid. This plasmid was then used as a template to amplify the CynD coding sequence with a C-terminal 6×His tag using the primers F_CynD and R_2_CynD (5′-tttGAATTCagtggtggtggtggtggtg-3′) and cloned in the NdeI and EcoRI sites of the pET-11 expression plasmid.

Recombinant CynD was expressed in the Escherichia coli BL21(DE3)/pLysS strain, induced by 0.3 mM isopropyl-β-d-1-thiogalactopyranoside for 23 h at 18°C. The cells obtained from 1 L of culture were lysed by sonication using 50 mL of lysis buffer (100 mM Tris-HCl [pH 8.0], 100 mM NaCl, and 50 mM imidazole), and the suspension was clarified by centrifugation (13,000 × *g*) for 45 min. The supernatant was loaded onto Ni-NTA affinity resin (His-trap chelating 5-mL column), washed with 10 column volumes of lysis buffer, and eluted with a gradient of 50 to 500 mM imidazole in 20 mM Tris-HCl (pH 8.0) and 100 mM NaCl. The eluted fractions were further purified by size exclusion chromatography using a Superdex pg 200 16/600 column and 20 mM Tris-HCl (pH 8.0) and 100 mM NaCl as the running buffer. The eluted fractions were evaluated for purity by SDS-PAGE, and fractions containing pure protein were concentrated in Amicon Ultra-15 centrifugal filter units.

### Enzymatic assays of recombinant CynD.

Enzymatic activity assays of recombinant CynD were performed in triplicate in Corning Costar 96-well polystyrene microplates at pH 8.0 at 30°C. An ammonia assay kit (Sigma-Aldrich, AA0100) was used to measure ammonia production. Cyanide volatilization was avoided by sealing the plate with a lid of the same material as the microplate. The CynD concentration was measured by determining the absorbance at 280 nm, assuming an extinction coefficient of 60,655 M^−1 ^cm^−1^. Reaction mixtures containing initial cyanide concentrations of 0, 0.39, 0.625, 0.78, 1.25, 1.56, 2.5, 3.125, 5, 6.25, 12.5, and 25 mM were prepared by adding 5 μL of NaCN stock solutions at 0, 8.67, 13.88, 17.34, 27.75, 34.69, 55.5, 69.38, 111, 138.75, 277.5, and 555 mM, respectively, to microplate wells containing 106 μL of 500 nM CynD in 100 mM NaCl and 100 mM Tris-HCl (pH 8). Measurements were recorded on a microplate reader (SpectraMax Paradigm; Molecular Devices) at 340 nm every 20 s. Calculations of ammonia concentrations were carried out according to the ammonia assay kit instructions. Ammonia production versus time was adjusted to a linear equation. The first three measurements (1-min total time) gave linear relationships, and the slopes were used to calculate initial velocities that were then divided by the total enzyme concentration (500 nM) to obtain the initial velocities (*V*_0_) expressed as the number of reactions per second per enzyme molecule at each substrate concentration. Initial velocities were plotted versus the initial cyanide concentration, and the *K_m_* and *V*_max_ values were estimated by using nonlinear least squares implemented in the R software (nls function in R).

To analyze the pH dependence of CynD activity, 5-μL portions of stock solutions of recombinant CynD at 0, 50, 100, 150, or 200 μM in 100 mM NaCl–Tris-HCl (pH 8) were added to 45 μL of reagent solution (40 mM NaCN, 100 mM NaCl, and 200 mM Tris-HCl or *N*-cyclohexyl-3-aminopropanesulfonic acid [CAPS]). Tris-HCl was used for reactions at pH 8 and 9, and CAPS was used for reactions at pH 10 and 11. This produces reactions with a final CynD concentration of 0, 5, 10, 15, or 20 μM and an initial cyanide concentration of 36 mM. The reaction mixtures were incubated for 10 min at 30°C. Subsequently, 100 μL of picric acid 5 mg/mL–0.25 M Na_2_CO_3_ was added, and the reaction mixtures were incubated at 99°C for 6 min. Next, 30 μL of this reaction was transferred to a 96-well microplate, and the absorbance at 520 nm was measured. The final cyanide concentration was estimated based on calibration curves with fresh NaCN solutions at concentrations between 0 and 40 mM that were analyzed in parallel with the enzymatic reactions under the same conditions.

### SEC-MALS.

SEC-MALS analysis was used to determine the oligomeric state of recombinant CynD. Molar mass analysis was done in 100 mM NaCl and 20 mM Tris-HCl or CAPS at pH 8, 9, or 10, 11, respectively. Protein samples (100-μL injection of 3.47 mg/mL [89.36 μM] CynD) were separated by using a Superdex 200 increase 10/300 GL coupled to a MiniDAWN TREOS multiangle light scattering system and an Optilab rEX refractive index detector. Data analysis was performed using the Astra Software package, version 7.1.1 (Wyatt Technology Corp.).

### Transmission electron microscopy.

Ultrathin carbon layers on lacey carbon-coated copper grids were negatively charged by glow discharge of 25 s at 15 mA. Portions (4 μL) of purified recombinant CynD in 20 mM Tris-HCl (pH 8.0) and 100 mM NaCl at different concentrations (3.25 mg/mL or 1.625 mg/mL) were spotted onto the negatively charged carbon-coated copper grid for 1 min, followed by capillary action blotting. The grids were washed twice with Milli-Q water and then stained with 2% uranyl acetate for 30 s before blotting and air drying. Electron micrograph images were obtained using a JEOL JEM 2100 transmission electron microscope operating at 200 kV and equipped with a Gatan ORIUS CCD detector.

### RT-qPCR to evaluate *in vivo* induction of *cynD* by cyanide.

The selected *Bacillus* strain was grown in meat broth (meat extract, 1 g/L; yeast extract, 2 g/L; peptone, 5 g/L; NaCl, 5 g/L; and MnCl_2_, 10 mg/L) during 12 h at 30°C and 200 rpm. Meat broth and MnCl_2_ have been reported to stimulate cyanide-degrading capacity of Bacillus pumilus (41). A portion (1 mL) of the culture was centrifuged at 500 × *g* for 1 min, and the supernatant was transferred to a clean 1.5-mL capped microcentrifuge tube and centrifuged at 8,000 × *g* for 3 min. The pellet was resuspended in 1 mL of 3.84 mM NaCN (equivalent to 100 ppm [CN^–^] or 188 ppm NaCN) in Milli-Q water. Controls were resuspended in 1 mL of Milli-Q water without NaCN. The tubes were maintained without agitation at 30°C for 4 h, and 100-μL portions were retrieved to measure the cyanide concentration by the picric acid method described above (42). A 900-μL sample was centrifuged, and the bacterial pellet was used immediately for total RNA extraction.

Total RNA extraction was done using TRIzol-chloroform protocol. Briefly, bacterial pellets were treated with 100 μL of lysozyme 3 mg/mL at 37°C for 30 min, and extraction was performed using a 5:1 TRIzol-chloroform mixture. After the extraction, the phase containing RNA was separated, and the RNA was precipitated using isopropanol. The RNA pellet was washed twice with 75% ethanol and finally resuspended in 20 μL of Tris 20 mM-DEPC. The total RNA concentration and purity were estimated in a NanoDrop One/OneC Microvolume UV-Vis spectrophotometer (Thermo Fisher Scientific), and the integrity was evaluated in a 2100 Bioanalyzer using an Agilent RNA 6000 Pico chip. After DNase treatment, the samples were subjected to PCR to verify the absence of DNA contamination. cDNA synthesis was performed with 1 μg of the RNA and a Thermo Scientific H minus first strand cDNA synthesis kit. cDNA synthesis was verified by PCR and electrophoresis.

Amplification efficiency of the primers used in the RT-qPCR were verified using 300 nM concentrations of each primer and a 2-fold dilution series of the cDNA to generate a standard curve composed of four concentrations: 62.5, 31.25, 15.625, and 7.8125 ng/μL. Each dilution reaction was performed in triplicate using a Maxima SYBR green/ROX qPCR Master Mix kit (Thermo Fisher Scientific) according to the manufacturer’s instructions in QuantStudio 3 equipment (Thermo Fisher Scientific). Primers for the normalizing gene *rpsJ* (F_rpsJ [5′-TGAAACGGCTAAGCGTTCTG-3′] and R_rpsJ [5′-ACGCATCTCGAATTGCTCAC-3′]) and for the nitrilases *cynD* (F_cynD [5′-TGCCCAAAATGAGCAGGTAC-3′] and R_cynD [5′-AAATGTCTGTGTCGCGATGG-3′]) and *EGI07_17510* (F_ *EGI07_17510* [5′-TTGGTGCGATGATTTGCTAT-3′] and R_ *EGI07_17510* [5′-GTGTCTCTGCTTGTGCCTGT-3′]) were tested for efficiency. The amplification efficiency of the qPCR was calculated through the slope of the cDNA curve obtained for each primer pair.

Since the primer pairs showed similar efficiencies (*EGI07_17510 *=* *119.108%, *cynD *=* *108.385%, and *rpsJ *=* *104.55%), we performed each qPCR assay in technical triplicates using 15.625 ng/μL of cDNA and the kit Maxima SYBR green/ROX qPCR Master Mix (Thermo Fisher Scientific) in QuantStudio 3 equipment (Thermo Fisher Scientific). ΔΔ*C_T_* values were calculated in the absence or presence of cyanide for the nitrilase genes *EGI07_17510* and *cynD* using *rpsJ* as the normalizing gene. Three biological replicates were performed.

### Data availability.

The final genome assembly is available in the IMG/M (29) and GenBank/NCBI (24) databases under accession numbers 2818991268 and RSEW00000000.1, respectively.
