# Supplementary material for: Genomic Characterization of Bacillus safensis Isolated from Mine Tailings in Peru and Evaluation of Its Cyanide-Degrading Enzyme CynD
Source: Appl Environ Microbiol. 2022 Jun 28;88(14):e00916-22. doi: 10.1128/aem.00916-22 (PMC9317851; doi:10.1128/aem.00916-22)
Supplement: Supplemental file 1 — Fig. S1 to S5 and Tables S1 to S6. Download aem.00916-22-s0001.pdf, PDF file, 1.7 MB [file aem.00916-22-s0001.pdf]

Genomic characterization of Bacillus safensis isolated from mine tailings in Peru and evaluation of its cyanide-degrading enzyme CynD

Santiago Justo Arevalo*[1,2], Daniela Zapata Sifuentes[1,2], Andrea Cuba Portocarrero[1], Michella Brescia Reategui[1], Claudia Monge Pimentel[1], Layla Farage Martins[2], Paulo Marques Pierry[2], Carlos Morais Piroupo[2], Alcides Guerra Santa Cruz[1], Mauro Quiñones Aguilar[1], Chuck Shaker Farah[2], João Carlos Setubal[2], Aline Maria da Silva[2]

1.- Facultad de Ciencias Biológicas, Universidad Ricardo Palma, Lima, Peru.

2.- Departamento de Bioquímica, Instituto de Química, Universidade de São Paulo, São Paulo, Brazil.

Supporting Information

Contents:

Figure S1. Map from the site of collection. The map was extracted from google earth. Yellow mark indicates where the sample was collected

Figure S2. Core genome identity matrix to classified genomes of *Bacillus pumilus* group genomes. An identity matrix of 132 core genomes of *Bacillus pumilus* group showing delimitations between three species: *Bacillus altitudinis* (brown names), *Bacillus safensis* (green names), *Bacillus pumilus* (blue names). Two core genomes (red names) appear outside of these three species.

Figure S3. Alignment of identical protein group CynDs with $CynD_{PER\text{-}URP\text{-}08}$ and $CynD_{C1}$. Homology of CynD from *Bacillus safensis* PER-URP-08 is clearly shown in the protein sequence alignments of several CynD homologs including those with tested enzymatic activity as CynD from *B. pumilus* strain C1. $CynD_{C1}$ and $CynD_{PER\text{-}URP\text{-}08}$ are highlighted in bold and by a red square.

Figure S4. Purification of recombinant $CynD_{PER\text{-}URP\text{-}08}$. SDS-PAGE showing the expression and purification of recombinant $CynD_{PER\text{-}URP\text{-}08}$.

Figure S5. $CynD_{PER\text{-}URP\text{-}08}$ production of NH_4^+ by time. Linear adjust of the product formation (NH_4^+) by CynD in the first 40 seconds of reaction using different initial concentrations of cyanide.

SUPLEMENTARY TABLES:

Table S1. Accession numbers of reference genomes used in the assembly process.
Table S2. Initial screening of cyanide degradation of 20 cyanide-resistant colonies
Table S3. BLAST best-hits of the partial 16S rRNA gene for each tested strain.
Table S4. Summary of IMG/M annotations of the *Bacillus safensis* PER-URP-08 genome.
Table S5. Summary information of the 132 genomes used in the core genomes analysis.
Table S6. Identical protein groups (IPG) NCBI accession IDs by strain and species.

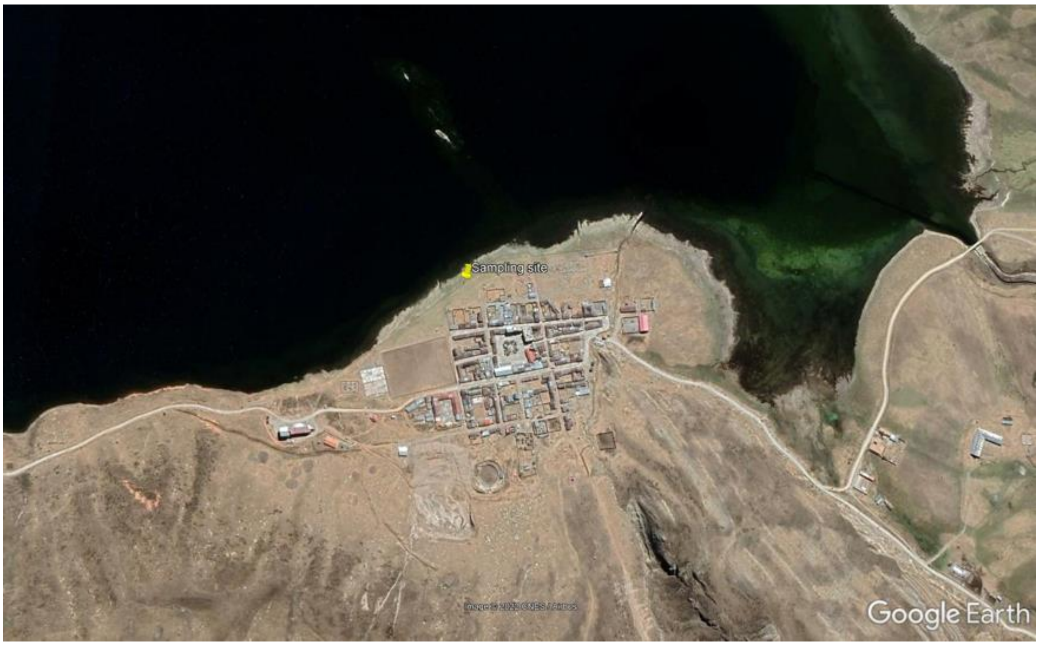

Figure S1. Map from the site of collection. The map was extracted from google earth. Yellow mark indicates where the sample was collected

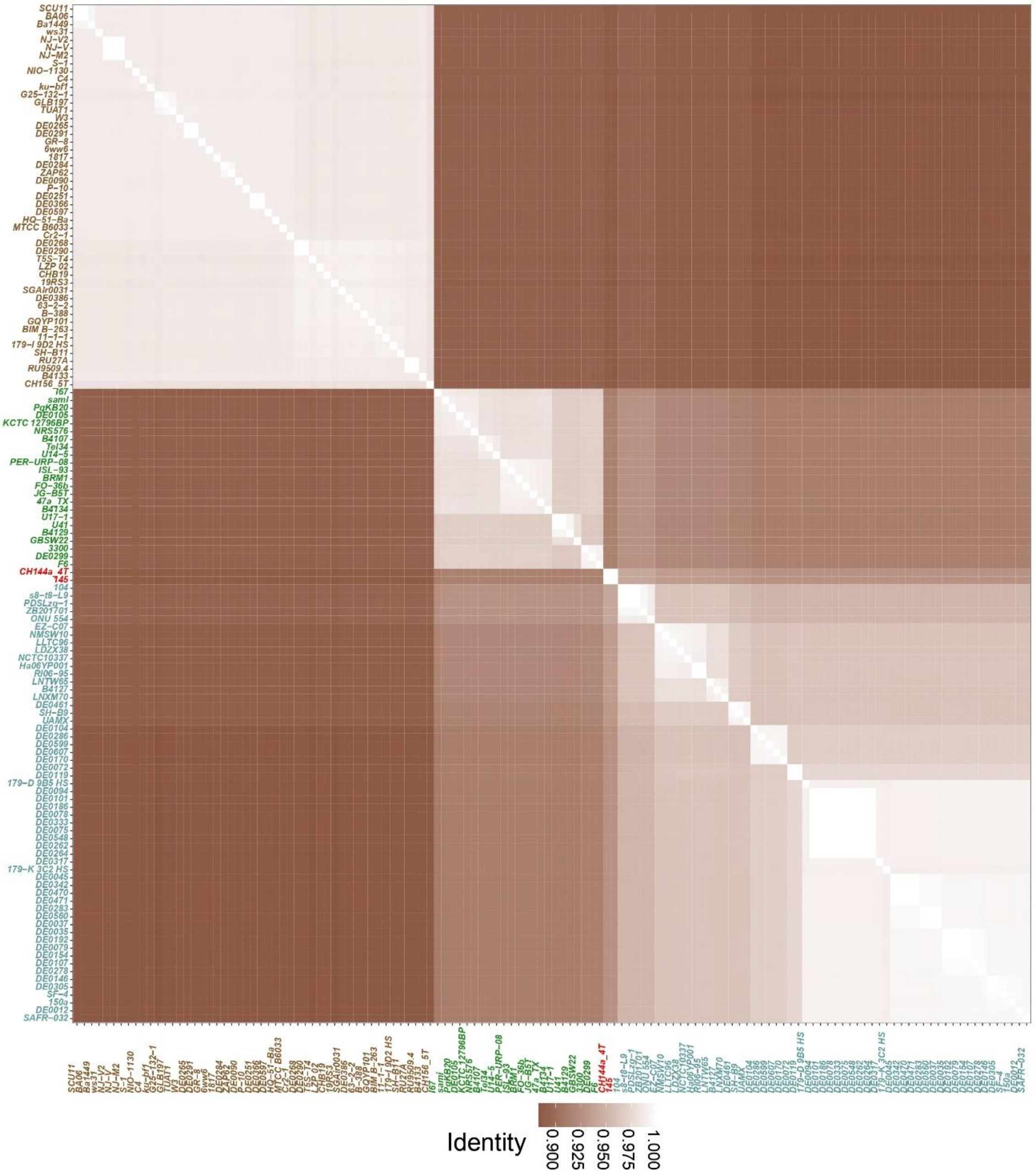

Figure S2. Core genome identity matrix to classified genomes of *Bacillus pumilus* group genomes. An identity matrix of 132 core genomes of *Bacillus pumilus* group showing delimitations between three species: *Bacillus altitudinis* (brown names), *Bacillus safensis* (green names), *Bacillus pumilus* (blue names). Two core genomes (red names) appear outside of these three species.

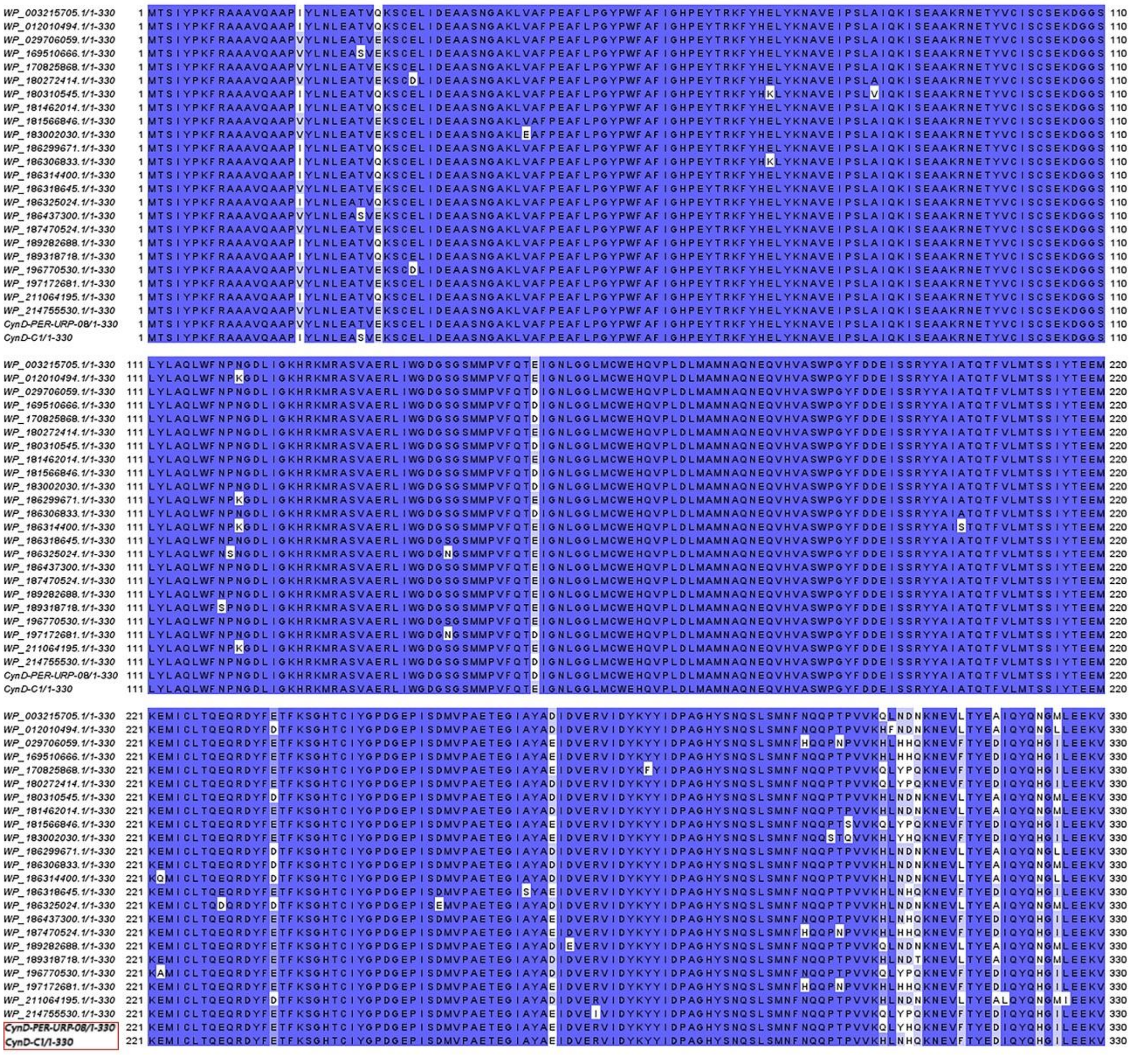

Figure S3. Alignment of identical protein group CynDs with CynD$_{PER\text{-}URP\text{-}08}$ and CynD$_{C1}$. Homology of CynD from *Bacillus safensis* PER-URP-08 is clearly shown in the protein sequence alignments of several CynD homologs including those with tested enzymatic activity as CynD from *B. pumilus* strain C1. CynD$_{C1}$ and CynD$_{PER\text{-}URP\text{-}08}$ are highlighted in bold and by a red square.

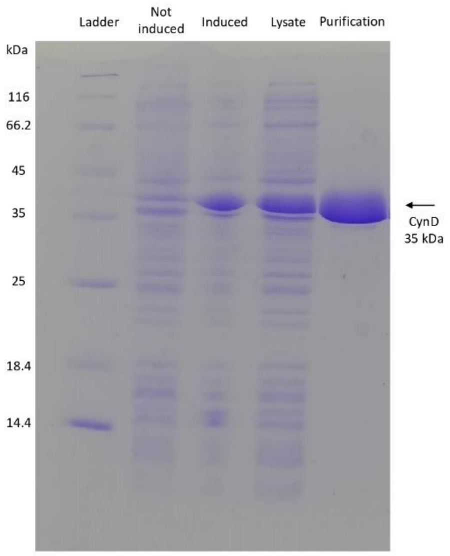

Figure S4. Purification of recombinant $CynD_{PER-URP-08}$. SDS-PAGE showing the expression and purification of recombinant $CynD_{PER-URP-08}$.

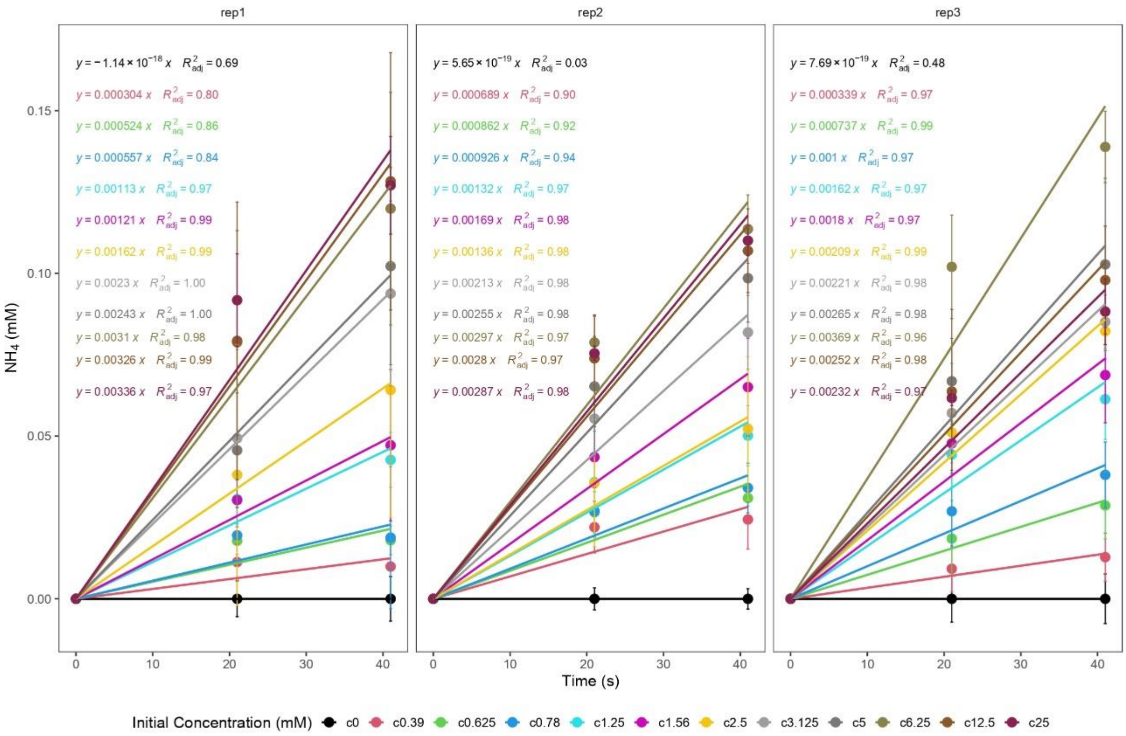

Figure S5. $CynD_{PER\text{-}URP\text{-}08}$ production of NH_4^+ by time. Linear adjust of the product formation (NH_4^+) by CynD in the first 40 seconds of reaction using different initial concentrations of cyanide.

Table S1. Accession numbers of reference genomes used in the assembly process.

Strain	IMG code
B. pumilus JRS3	2667527735
B. pumilus SAFR-032	640753007
B. pumilus TUAT1	2684623054
B. pumilus SH-B11	2687453109
B. pumilus RI06-95	2639762961

Table S2. Initial screening of cyanide degradation of 20 cyanide-resistant colonies

Strain	Cyanide removal (%)
1	19,45
2	4,11
3	10,41
4	35,82
5	37,9
6	42,15
7	1,27
8*	69,92
9	44,96
10	2,47
11	17,45
12	66,11
13	10,42
14	40,82
15	14,9
16	21,45
17	63,31
18	0,41
19	29,82
20	32,41

*Degradation assays with the strain with the highest percentage of cyanide degradation were repeated three times and reported in the main text

Table S3. BLAST best-hits of the partial 16S rRNA gene for each tested strain.

Accession ID	Description	Identity	E-value	Reference
MZ723096.1	Bacillus safensis strain LgS5 16S ribosomal RNA gene, partial sequence	100%	2,00E-166	Unpublished
MZ722995.1	Bacillus pumilus strain YG35 16S ribosomal RNA gene, partial sequence	100%	2,00E-166	Unpublished
MZ720806.1	Bacillus australimaris strain EPB15 16S ribosomal RNA gene, partial sequence	100%	2,00E-166	Unpublished
MZ720801.1	Bacillus safensis strain EPB9 16S ribosomal RNA gene, partial sequence	100%	2,00E-166	Unpublished
MZ707643.1	Bacillus pumilus strain XY36 16S ribosomal RNA gene, partial sequence	100%	2,00E-166	Unpublished

Table S4. Summary of IMG/M annotations of the *Bacillus safensis* PER-URP-08 genome.

**Strain**	*B. safensis* **PER-URP-08**
**Assembly**	
Coverage	694x
Number of contigs	17
Contig N50	3013666
Contig L50	1
Total base pairs	3718369
**Annotation**	
Total coding base pairs	3305799
G+C Percentage	41,61
Total Number of Genes	3872
Total Number of Protein-coding Genes	3758
RNA genes	114
rRNA genes	9
5s rRNA	7
16s rRNA	2
23s rRNA	0
tRNA genes	75
Other RNA genes	30
Proteins with predicted function	3197
Proteins without predicted function	651
Enzymes-coding genes	1054
Cromosomals cassettes	287
Genes of transmembrane proteins	1062

Table S5. Summary information of the 132 genomes used in the core genomes analysis.

Specie	GB_Specie	Strain	CynD_presence	Location	Assembly_ID
B. altitudinis	B. altitudinis	11-1-1	no	Belarus	GCA_013283915.1
	B. altitudinis	179-I 9D2 HS	no	USA	GCA_019037255.1
	B. altitudinis	1817	no	China	GCA_017161205.1
	B. altitudinis	19RS3	no	Argentina	GCA_013391605.1
	B. altitudinis	63-2-2	no	Belarus	GCA_016807685.1
	B. altitudinis	6ww6	no	China	GCA_017948365.1
	B. altitudinis	B-388	no	USA	GCA_000789425.2
	B. altitudinis	B4133	no	Netherlands	GCA_000828455.1
	B. altitudinis	BA06	no	China	GCA_000299555.2
	B. altitudinis	Ba1449	no	China	GCA_015689015.1
	B. altitudinis	BIM B-263	no	Belarus	GCA_015160895.1
	B. pumilus	C4	no	Egypt	GCA_001687085.1
	B. altitudinis	CH156_5T	no	Mexico	GCA_008180475.1
	B. altitudinis	CHB19	no	Malaysia	GCA_004563755.2
	B. altitudinis	Cr2-1	no	China	GCA_007923025.1
	B. altitudinis	DE0090	no	USA	GCA_007682105.1
	B. altitudinis	DE0251	no	USA	GCA_008764185.1
	B. altitudinis	DE0265	no	USA	GCA_007681425.1
	B. altitudinis	DE0268	no	USA	GCA_007681435.1
	B. altitudinis	DE0284	no	USA	GCA_007681345.1
	B. altitudinis	DE0290	no	USA	GCA_007681315.1
	B. altitudinis	DE0291	no	USA	GCA_007681245.1
	B. altitudinis	DE0366	no	USA	GCA_007676515.1
	B. altitudinis	DE0386	no	USA	GCA_007676435.1
	B. altitudinis	DE0597	no	USA	GCA_007671735.1
	B. altitudinis	G25-132-1	no	China	GCA_015846075.1
	B. altitudinis	GLB197	no	China	GCA_001908475.1
	B. altitudinis	GQYP101	no	China	GCA_005849435.1
	B. altitudinis	GR-8	no	China	GCA_001191605.1
	B. altitudinis	HQ-51-Ba	no	not_collected	GCA_006007905.1
	B. altitudinis	ku-bf1	no	India	GCA_001543165.1
	B. altitudinis	LZP 02	no	China	GCA_019164215.1
	B. pumilus	MTCC B6033	no	India	GCA_000590455.1
	B. altitudinis	NIO-1130	no	India	GCA_001457015.1
	B. altitudinis	NIO-1130	no	not_collected	GCA_900094985.1
	B. altitudinis	NJ-M2	no	China	GCA_001431145.1
	B. altitudinis	NJ-V	no	China	GCA_001700735.1
	B. altitudinis	NJ-V2	no	China	GCA_001431785.1
	B. altitudinis	P-10	no	Indonesia	GCA_002741745.1
	B. altitudinis	RU27A	no	not_collected	GCA_900188195.1
	B. altitudinis	RU9509.4	no	not_collected	GCA_900119345.1
	B. altitudinis	S-1	no	not_collected	GCA_000225935.1

	B. altitudinis	SCU11	no	China	GCA_013307105.1
	B. altitudinis	SCU11	no	China	GCA_019355135.1
	B. altitudinis	SGAir0031	no	Singapore	GCA_002443015.2
	B. pumilus	SH-B11	no	Netherlands	GCA_001578165.1
	B. altitudinis	T5S-T4	no	Argentina	GCA_013391615.1
	B. pumilus	TUAT1	no	Japan	GCA_001548215.1
	B. altitudinis	W3	no	China	GCA_000972685.1
	B. altitudinis	ws31	no	China	GCA_016767855.1
	B. altitudinis	ZAP62	no	Mexico	GCA_011067205.1
B. pumilus	B. pumilus	104	no	USA	GCA_003034105.1
	B. pumilus	DE0104	no	USA	GCA_007679395.1
	B. pumilus	DE0170	no	USA	GCA_007678395.1
	B. pumilus	DE0286	no	USA	GCA_007676815.1
	B. pumilus	DE0599	no	USA	GCA_007665445.1
	B. pumilus	DE0607	no	USA	GCA_007665325.1
	B. pumilus	Ha06YP001	no	USA	GCA_003020795.1
	B. pumilus	ONU 554	no	Ukraine	GCA_014489355.1
	B. pumilus	PDSLzg-1	no	China	GCA_001704975.1
	B. pumilus	RI06-95	no	USA	GCA_001183525.1
	B. pumilus	s8-t8-L9	no	Atlantic Ocean	GCA_018128785.1
	B. pumilus	ZB201701	no	China	GCA_004006455.1
	B. pumilus	150a	yes	Mexico	GCA_003571425.1
	B. pumilus	179-D 9B5 HS	yes	USA	GCA_019037765.1
	B. pumilus	179-K 3C2 HS	yes	USA	GCA_019036865.1
	B. pumilus	B4127	yes	Netherlands	GCA_000828345.1
	B. pumilus	DE0012	yes	USA	GCA_007680695.1
	B. pumilus	DE0035	yes	USA	GCA_007680335.1
	B. pumilus	DE0037	yes	USA	GCA_007680315.1
	B. pumilus	DE0045	yes	USA	GCA_007680195.1
	B. pumilus	DE0072	yes	USA	GCA_007679805.1
	B. pumilus	DE0075	yes	USA	GCA_007679755.1
	B. pumilus	DE0078	yes	USA	GCA_007679665.1
	B. pumilus	DE0079	yes	USA	GCA_007679675.1
	B. pumilus	DE0094	yes	USA	GCA_007679515.1
	B. pumilus	DE0101	yes	USA	GCA_007679485.1
	B. pumilus	DE0107	yes	USA	GCA_007679415.1
	B. pumilus	DE0119	yes	USA	GCA_007679215.1
	B. pumilus	DE0146	yes	USA	GCA_007678815.1
	B. pumilus	DE0154	yes	USA	GCA_007678705.1
	B. pumilus	DE0186	yes	USA	GCA_007678135.1
	B. pumilus	DE0192	yes	USA	GCA_007678035.1
	B. pumilus	DE0262	yes	USA	GCA_007677155.1
	B. pumilus	DE0264	yes	USA	GCA_007677125.1
	B. pumilus	DE0278	yes	USA	GCA_007676935.1
	B. pumilus	DE0283	yes	USA	GCA_007676865.1

	B. pumilus	DE0305	yes	USA	GCA_007674165.1
	B. pumilus	DE0317	yes	USA	GCA_007674015.1
	B. pumilus	DE0333	yes	USA	GCA_007673765.1
	B. pumilus	DE0342	yes	USA	GCA_007673705.1
	B. pumilus	DE0461	yes	USA	GCA_007667685.1
	B. pumilus	DE0470	yes	USA	GCA_007667505.1
	B. pumilus	DE0471	yes	USA	GCA_007667475.1
	B. pumilus	DE0548	yes	USA	GCA_007666215.1
	B. pumilus	DE0560	yes	USA	GCA_007666085.1
	B. pumilus	EZ-C07	yes	Russia	GCA_003301255.1
	B. pumilus	LDZX38	yes	China	GCA_002998475.1
	B. pumilus	LLTC96	yes	China	GCA_002998365.1
	B. pumilus	LNTW65	yes	China	GCA_002998415.1
	B. pumilus	LNXM70	yes	China	GCA_002998395.1
	B. pumilus	NCTC10337	yes	not_collected	GCA_900186955.1
	B. pumilus	NMSW10	yes	China	GCA_002998335.1
	B. pumilus	SAFR-032	yes	not_collected	GCA_000017885.4
	B. pumilus	SF-4	yes	Pakistan	GCA_009937765.1
	B. pumilus	SH-B9	yes	Netherlands	GCA_001578205.1
	B. pumilus	UAMX	yes	Mexico	GCA_013423765.1
B. safensis	B. safensis	B4107	no	Netherlands	GCA_000828395.1
	B. safensis	NRS576	no	not_collected	GCA_900573445.1
	B. safensis	Tel34	no	Greece	GCA_016767355.1
	B. safensis	U14-5	no	Antarctica	GCA_001938665.1
	B. safensis	3300	yes	USA	GCA_007829795.1
	B. safensis	47a_TX	yes	USA	GCA_003610615.1
	B. safensis	B4129	yes	Netherlands	GCA_000828375.1
	B. safensis	B4134	yes	Netherlands	GCA_000828425.1
	B. safensis	BRM1	yes	Brazil	GCA_002077215.1
	B. safensis	DE0105	yes	USA	GCA_008764375.1
	B. safensis	DE0299	yes	USA	GCA_007674245.1
	B. safensis	F6	yes	Belarus	GCA_016803835.1
	B. safensis	FO-36b	yes	USA	GCA_003097715.1
	B. safensis	GBSW22	yes	China	GCA_002998315.1
	B. safensis	I67	yes	Brazil	GCA_012972765.1
	B. safensis	ISL-93	yes	Chile	GCA_018614995.1
	B. safensis	JG-B5T	yes	Germany	GCA_003284765.1
	B. safensis	KCTC 12796BP	yes	South Korea	GCA_001895885.1
	B. pumilus	PER-URP-08	yes	Peru	GCA_016629615.1
	B. safensis	PgKB20	yes	South Korea	GCA_008244765.1
	B. safensis	sami	yes	Pakistan	GCA_003660145.1
	B. safensis	U17-1	yes	Antarctica	GCA_001938705.1
	B. safensis	U41	yes	Antarctica	GCA_001938685.1
B. sp	B. pumilus	145	no	Mexico	GCA_003431975.1
	B. pumilus	CH144a_4T	no	Mexico	GCA_008180455.1

Table S6. Identical protein groups (IPG) NCBI accession IDs by strain and species.

IPG	Strain	Specie
WP_003215705.1	LLTC96	*B. pumilus*
	NCTC10337	
WP_012010494.1	SAFR-032	
WP_180310545.1	UAMX	
WP_181462014.1	B4127	
	LNTW65	
WP_186299671.1	150a	
	179-D 9B5 HS	
	DE0012	
	DE0037	
	DE0045	
	DE0075	
	DE0078	
	DE0094	
	DE0101	
	DE0146	
	DE0186	
	DE0262	
	DE0264	
	DE0283	
	DE0305	
	DE0317	
	DE0333	
	DE0342	
	DE0470	
	DE0471	
	DE0548	
	DE0560	
	SF-4	
WP_186306833.1	DE0461	
	SH-B9	
WP_186314400.1	DE0035	
	DE0079	
	DE0107	
	DE0154	
	DE0192	
	DE0278	
WP_186325024.1	DE0072	
	DE0119	
WP_189282688.1	EZ-C07	
	LDZX38	
	NMSW10	

WP_189318718.1	LNXM70	
WP_211064195.1	179-K 3C2 HS	
PER-URP-08	PER-URP-08	*B. safensis*
WP_029706059.1	DE0105	
	KCTC 12796BP	
WP_169510666.1	I67	
WP_170825868.1	B4134	
	BRM1	
	FO-36b	
WP_180272414.1	B4129	
	GBSW22	
WP_181566846.1	JG-B5T	
WP_183002030.1	47a_TX	
WP_186318645.1	DE0299	
	F6	
WP_186437300.1	3300	
WP_187470524.1	PgKB20	
WP_196770530.1	U17-1	
	U41	
WP_197172681.1	sami	
WP_214755530.1	ISL-93	
